# The dead lithium formation under mechano-electrochemical coupling in lithium metal batteries

**DOI:** 10.1016/j.fmre.2022.11.005

**Published:** 2022-11-23

**Authors:** Xin Shen, Rui Zhang, Peng Shi, Xue-Qiang Zhang, Xiang Chen, Chen-Zi Zhao, Peng Wu, Yi-Ming Guo, Jia-Qi Huang, Qiang Zhang

**Affiliations:** a Beijing Key Laboratory of Green Chemical Reaction Engineering and Technology, Department of Chemical Engineering, Tsinghua University, Beijing 100084, China; b Advanced Research Institute of Multidisciplinary Science, Beijing Institute of Technology, Beijing 100081, China; c School of Materials Science and Engineering, Beijing Institute of Technology, Beijing 100081, China; d Research & Development Center BMW, BMW China Services Ltd., Beijing 101318, China

**Keywords:** Stress-coupled stripping, Li dendrites, Dead Li, Li metal batteries, Phase field model simulation

## Abstract

Lithium metal is one of the most promising anode materials for next-generation high-energy-density rechargeable batteries. A fundamental mechanism understanding of the dead lithium formation under the interplay of electrochemistry and mechanics in lithium metal batteries is strongly considered. Herein, we proposed a mechano-electrochemical phase-field model to describe the lithium stripping process and quantify the dead lithium formation under stress. In particular, the rupture of solid electrolyte interphase and the shift of equilibrium potential caused by stress are coupled into stripping kinetics. The impact of external pressure on dead lithium formation with various electrolyte properties and initial electrodeposited morphologies is revealed. The overlooked detrimental effect of external pressure on Li stripping affords fresh insights into cell configuration and pressure management, which is critical for practical applications of lithium metal batteries.

## Graphical abstract

**Figure:**
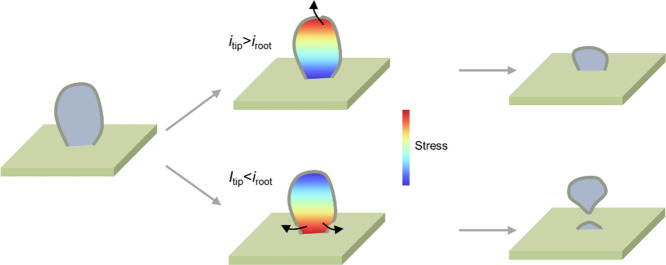

## 1 Introduction

The explosive growth of electrochemical energy storage demands, especially in electric vehicles and consumer electronics, continuously stimulates rechargeable batteries to upgrade and stride forward in the energy density. Lithium (Li) metal is a promising anode material for next-generation high-energy-density batteries because of its ultrahigh theoretical specific capacity (3860 mAh g^−1^) and low redox potential (−3.04 V versus the standard hydrogen potential electrode) [1]. The energy density of the corresponding Li metal batteries enables a step-change improvement compared to that of current Li-ion batteries [2,3]. However, the uncontrollable emergence of Li dendrites on working Li metal anodes can penetrate the separator to short the batteries, going along with capacity loss from side reaction and dead Li formation [4,5]. Besides, Li metal anodes undergo infinite volume change due to conversion reactions, making the mechanical issues more pronounced compared to routine graphite anodes [6]. The inferior performance and safety hazards caused thereby remarkably hinder the implementation of Li metal batteries.

Unlocking the mechanism of Li dendrites and manipulating the plating/stripping behavior of Li in turn are critical for rooting out the issues in Li metal anodes. A significant body of researchers has investigated the plating process of Li through models and simulations [7,8]. In terms of the origin of dendrites, Chazalviel et al. proposed a space-charge model that the onset of ramified metallic electrodeposits is caused by the creation of a space charge upon ion depletion at the electrode vicinity [9,10], where the transition time is characterized by Sand's time [11,12]. In contrast, the surface self-diffusion model implies that dendrite growth is an inherent property of Li metal [13,14]. Recently, time-dependent models incorporating the above factors have been built with the development of interface reconstruction methods, such as phase field [15,16], kinetic Monte Carlo [17], level set [18], and deformed mesh [19], further quantifying the ion/atom transport and overpotential dependence on morphologic evolution. Specifically, the introduction of the stress coupling effect highlights the practical concern of Li metal batteries in various applications, reinforcing the reliability of model-guided design [20]. For instance, Newman et al. incorporated the internal stress coming from solid-solid contact (Li/polymer interface) into a kinetic model and demonstrated that the separator with a shear modulus twice that of lithium can suppress dendrites [21]. We described how external pressure impacts the dendrites, aligning academic research (coin cells) with industrial applications (pouch cells) [22]. Experiments performed by Louli et al. verify that suitable pressure can improve the electrochemical performance of lithium metal anodes [23]. However, the fundamental investigation into the complex process between the Li strips and dead Li forms is few touched yet.

Electrodeposited Li could be transferred back to Li ions theoretically by a reverse stripping process. Unfortunately, dead Li—which is electrically isolated from an electrode and represents an irreversible Li loss—arises as a result of inhomogeneous electrochemical reactions and mechanical effects. As cycling proceeds, its accumulation can further block ion transport and Li stripping, causing a self-accelerating positive feedback deterioration of batteries [24], [25], [26]. Such qualitative understanding of Li stripping has been verified by experimental observation based on various *in/ex-situ* microscopies [27,28]. With the application of titration gas chromatography, the composition of dead Li (hollowed-out solid electrolyte interphase (SEI) and Li metal debris electronically isolated by SEI) is distinguished and quantified [29,30]. Iodine redox is proposed for effectively rejuvenating the dead Li based on the interplay mechanism between dead Li and Li stripping behaviors [31,32]. However, very little is known about how mechanics are intertwined with electrochemistry in the stripping process, which is urgently needed to quantitatively regulate the dead Li formation.

In this contribution, the Li stripping process with dead Li formation is investigated by a fully coupled mechano-electrochemical phase field model, where stress-induced potential shift and SEI fracture are considered. The influence of external pressure upon a wide range of electrolyte properties and working conditions on dead Li formation is revealed quantificationally. The potentially detrimental effect of external pressure indicates that separately managing the pressure during charging and discharging processes is required for achieving high-performance Li metal batteries.

## 2 Materials and methods

### 2.1 Theoretical framework

#### 2.1.1 Stress-coupled electrode reactions

**Fig. 1:**
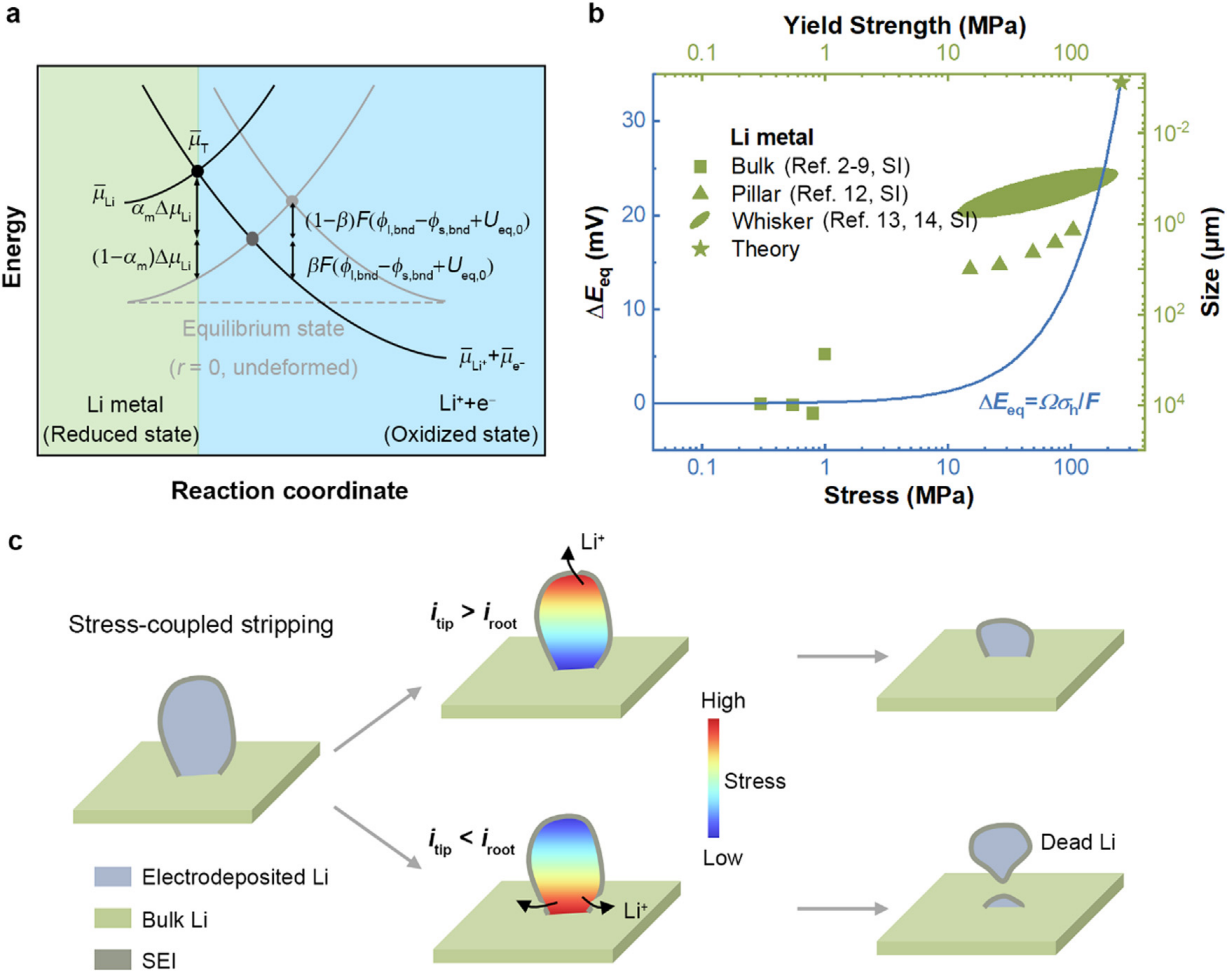
**Stress-coupled stripping in Li metal anodes.** (a) Energy diagram for the electrochemical reaction under stress. The light gray line indicates the energy landscape at the equilibrium state; The dark gray line represents an electric contribution; The black line denotes the mechanical contribution. Detailed symbolic descriptions can be seen in methods. (b) The deviation of equilibrium potential under stress and the yield strength of Li metal with various sizes. (c) Schematic of two scenarios caused by stress-coupled stripping.

For the electrochemical reaction across the Li–electrolyte interface:

(1) $$\text{Li}⇌Li^{+}+e^{-}$$

its energy landscape is shown schematically in Fig. 1a, describing an electron transfer at a transition state. The overall thermodynamic driving force can be described as

(2) $$\Delta G=\left({\bar{\mu }}_{T}-{\bar{\mu }}_{\text{Li}}\right)+\left({\bar{\mu }}_{Li^{+}}+{\bar{\mu }}_{e^{-}}-{\bar{\mu }}_{T}\right)$$

where ${\bar{\mu }}_{i}$ represents the electrochemical potential of species i (Li, Li^+^, *e*^−^, T, represents Li metal, Li ions, electron, and transition state species, respectively). Combined with the Arrhenius equation, the net rate of reaction r can be written as

(3) $$r=r_{a}-r_{c}=r_{0}\text{exp}\left(-\frac{{\bar{\mu }}_{T}-{\bar{\mu }}_{\text{Li}}}{RT}\right)-r_{0}\text{exp}\left[-\frac{{\bar{\mu }}_{T}-\left({\bar{\mu }}_{Li^{+}}+{\bar{\mu }}_{e^{-}}\right)}{RT}\right]$$

where $r_{0}$ is a rate constant, $r_{a}$ the rate of anodic reaction (stripping), and $r_{c}$ the cathodic reaction (plating). These electrochemical potentials can be further given by

(4) $${\bar{\mu }}_{\text{Li}}=\mu_{\text{Li}}^{\Theta }+RT\text{ln}a_{\text{Li}}+\Delta \mu_{\text{Li}}$$

(5) $${\bar{\mu }}_{Li^{+}}=\mu_{Li^{+}}^{\Theta }+RT\text{ln}a_{Li^{+}}+F\phi_{l,\text{bnd}}$$

(6) $${\bar{\mu }}_{e^{-}}=-F\phi_{s,\text{bnd}}$$

(7) $${\bar{\mu }}_{T}=\mu_{T}^{\Theta }+\left(1-\beta \right)F\left(\phi_{l,\text{bnd}}-\phi_{s,\text{bnd}}\right)+\alpha_{m}\Delta \mu_{\text{Li}}$$

where $\mu_{i}^{\Theta }$ is the standard state chemical potential of species i, $a_{i}$ the activity of species i, $\phi_{l,\text{bnd}}$ the electric potential of electrolyte near the interface, $\phi_{s,\text{bnd}}$ the electric potential of solid near the interface, $\Delta \mu_{\text{Li}}$ the shift in the chemical potential because of the mechanical deformation in the Li metal electrode, *R* the ideal gas constant, *T* the temperature, and F Faraday's constant. The cathodic symmetry factor β represents the fraction of the applied potential which promotes the cathodic reaction, while the anodic symmetry factor $\alpha_{m}$ represents the fraction of the applied stress which promotes the anodic reaction. We assumed for the sake of simplicity that the liquid electrolyte stored in a separator is free of stress, thus the electrochemical potential of Li ions is independent of stress. Substituting Eqs. 4–7 into Eq. 3 yields

(8) $$\begin{array}{ccc} r_{a} & = & r_{0}\text{exp}\left(-\frac{\mu_{T}^{\Theta }-\mu_{\text{Li}}^{\Theta }}{RT}\right)a_{\text{Li}}\exp\left[\frac{\left(1-\beta \right)F\left(\phi_{s,\text{bnd}}-\phi_{l,\text{bnd}}\right)}{RT}\right]\\ & & \times \;\exp\left[\frac{\left(1-\alpha_{m}\right)\Delta \mu_{\text{Li}}}{RT}\right] \end{array}$$

(9) $$r_{c}=r_{0}\text{exp}\left(-\frac{\mu_{T}^{\Theta }-\mu_{Li^{+}}^{\Theta }}{RT}\right)a_{Li^{+}}\text{exp}\left[-\frac{\beta F\left(\phi_{s,\text{bnd}}-\phi_{l,\text{bnd}}\right)}{RT}\right]\text{exp}\left(-\frac{\alpha_{m}\Delta \mu_{\text{Li}}}{RT}\right)$$

With the definition of anodic rate constants $k_{a}$ and cathodic rate constants $k_{c}$ defined by

(10) $$k_{a}=r_{0}\text{exp}\left(-\frac{\mu_{T}^{\Theta }-\mu_{\text{Li}}^{\Theta }}{RT}\right)$$

(11) $$k_{c}=r_{0}\text{exp}\left(-\frac{\mu_{T}^{\Theta }-\mu_{Li^{+}}^{\Theta }}{RT}\right)$$

the net reaction rate becomes

(12) $$\begin{array}{ccc} r & = & k_{a}a_{\text{Li}}\exp\left[\frac{\left(1-\beta \right)F\left(\phi_{s,\text{bnd}}-\phi_{l,\text{bnd}}\right)}{RT}\right]\exp\left[\frac{\left(1-\alpha_{m}\right)\Delta \mu_{\text{Li}}}{RT}\right]\\ & & -\;k_{c}a_{Li^{+}}\text{exp}\left[-\frac{\beta F\left(\phi_{s,\text{bnd}}-\phi_{l,\text{bnd}}\right)}{RT}\right]\text{exp}\left(-\frac{\alpha_{m}\Delta \mu_{\text{Li}}}{RT}\right) \end{array}$$

When the net reaction rate is zero (r=0), the equilibrium potential $U_{\text{eq}}$ of the potential difference $\phi_{s,\text{bnd}}-\phi_{l,\text{bnd}}$ equals

(13) $$U_{\text{eq}}=E^{\Theta }+\frac{RT}{F}\ln\left(\frac{a_{Li^{+}}}{a_{\text{Li}}}\right)-\frac{\Delta \mu_{\text{Li}}}{F}$$

(14) $$E^{\Theta }=\frac{RT}{F}\ln\left(\frac{k_{c}}{k_{a}}\right)$$

Herein, $E^{\Theta }$ is the standard half-cell potential. We introduce $U_{\text{eq},\;\;\text{ud}}$ to denote the value of $U_{\text{eq}}$ at the undeformed state:

(15) $$U_{\text{eq},\;\;\text{ud}}=E^{\Theta }+\frac{RT}{F}\ln\left(\frac{a_{Li^{+}}}{a_{\text{Li}}}\right)$$

The charge transfer overpotential is defined as the difference between the actual potential difference and the equilibrium potential:

(16) $$\eta_{\text{ct}}=\phi_{s,\text{bnd}}-\phi_{l,\text{bnd}}-U_{\text{eq}}=\phi_{s,\text{bnd}}-\phi_{l,\text{bnd}}-U_{\text{eq},\text{ud}}+\frac{\Delta \mu_{\text{Li}}}{F}$$

We can further divide the charge transfer overpotential into the sum of activation-dependent overpotential $\eta_{\text{cta}}$ and concentration-dependent overpotential $\eta_{\text{ctc}}$:

(17) $$\eta_{\text{cta}}=\phi_{s,\text{bnd}}-\phi_{l,\text{bnd}}-E^{\Theta }+\frac{\Delta \mu_{\text{Li}}}{F}$$

(18) $$\eta_{\text{ctc}}=-\frac{RT}{F}\ln\left(\frac{a_{Li^{+}}}{a_{\text{Li}}}\right)$$

Eliminating $\phi_{s,\text{bnd}}-\phi_{l,\text{bnd}}$ using Eqs. 13–18 and combining the proportional relation between the local current density $i_{\text{loc}}$ and the net rate of reaction r give

(19) $$i_{\text{loc}}=rF=i_{0}\left\{\text{exp}\left[\frac{\left(1-\beta \right)F\eta_{\text{cta}}}{RT}\right]-\frac{a_{Li^{+}}}{a_{\text{Li}}}\text{exp}\left(-\frac{\beta F\eta_{\text{cta}}}{RT}\right)\right\}$$

where the exchange current density $i_{0}$ is defined by

(20) $$i_{0}=Fk_{a}^{\beta }k_{c}^{1-\beta }a_{\text{Li}}\text{exp}\left[\frac{\left(\beta -\alpha_{m}\right)\Delta \mu_{\text{Li}}}{RT}\right]$$

The exchange current density at the undeformed state is defined as

(21) $$i_{0,\text{ud}}=Fk_{a}^{\beta }k_{c}^{1-\beta }a_{\text{Li}}$$

Finally, $\Delta \mu_{\text{Li}}$ simplified from the equation derived from Monroe and Newman [21] is taken to be

(22) $$\Delta \mu_{\text{Li}}=\Omega \sigma_{h}$$

where Ω is the molar volume of the lithium, and $\sigma_{h}$ the hydrostatic stress.

In view of the reaction acceleration brought by SEI fracture, a correction factor *C* is introduced into Eq. 22 [33]:

(23) $$\Delta \mu_{\text{Li}}=C\Omega \sigma_{h}$$

In summary, the local current density can be described by

(24) $$\begin{array}{ccc} i_{\text{loc}} & = & i_{0,\text{ud}}\text{exp}\left[\frac{\left(\beta -\alpha_{m}\right)C\Omega \sigma_{h}}{RT}\right]\\ & & \times \;\left\{\exp\left[\frac{\left(1-\beta \right)F\eta_{\text{cta}}}{RT}\right]-\frac{c_{Li^{+}}}{c_{\text{Li}}}\text{exp}\left(-\frac{\beta F\eta_{\text{cta}}}{RT}\right)\right\} \end{array}$$

(25) $$\eta_{\text{cta}}=\phi_{s,\text{bnd}}-\phi_{l,\text{bnd}}-E^{\Theta }+\frac{C\Omega \sigma_{h}}{F}$$

#### 2.1.2 Phase-field equation

An order parameter ξ is introduced to distinguish the phase, where ξ=0 represents the electrolyte phase, and ξ=1 denotes the lithium metal phase. The interface between lithium metal electrode and electrolyte can be represented by the region where 0<ξ<1. The total Gibbs free energy of the system is given by

(26) $$G=\int_{V}\left[f_{\text{grad}}\left(\xi \right)+f_{\text{ch}}\left(\xi ,c_{i}\right)+f_{\text{elec}}\left(\xi ,c_{i},\phi \right)+f_{\text{els}}\left(\xi ,u\right)\right]dV$$

where $f_{\text{grad}}\left(\xi \right)=0.5\kappa \nabla^{2}\xi$ represents the gradient energy density. The gradient coefficient κ can be given by $\kappa \left(\theta \right)=\kappa_{0}\left[1+\delta \cos(\omega \theta )\right]$, considering the anisotropy of Li metal. $\kappa_{0}$, δ, ω, and θ are the gradient energy coefficient constant, the anisotropy strength, the anisotropy mode, and the relative angle of the interface normal vector, respectively. $f_{ch}=g\left(\xi \right)+\sum c_{i}\left(RT\ln c_{i}+\mu_{i}^{\theta }\right)$ denotes the free energy density for the bulk. $g\left(\xi \right)=W\xi^{2}{\left(1-\xi \right)}^{2}$ is a double-well function, which describes the two equilibrium states for the Li metal electrode and the electrolyte. W/16 is the height of the energy barrier. $f_{\text{elec}}\left(\xi ,c_{i},\phi \right)=\sum c_{i}Fz_{i}\phi$ is the electrostatic energy density, where ϕ is the electric potential, F the Faraday's constant, and $z_{i}$ the valence of species i. $f_{\text{els}}\left(\xi ,u\right)=0.5C_{ijkl}\varepsilon_{ij}^{E}\varepsilon_{kl}^{E}$ is the mechanical energy density stemming from the elastic deformation. $C_{ijkl}$ is the local phase-dependent stiffness tensor and $\varepsilon_{ij}^{E}$ denotes the elastic strain tensor.

The temporal evolution of the phase driven by the charge transfer reaction can be described by

(27) $$\frac{\partial \xi }{\partial t}=-L_{\sigma }\frac{\partial G}{\partial \xi }-M_{\eta }h^{\prime }\left(\xi \right)i_{\text{loc}}$$

Herein $L_{\sigma }$ represents the interface mobility and $M_{\eta }$ is a rate constant. $h\left(\xi \right)=\xi^{3}\left(6\xi^{2}-15\xi +10\right)$ is an interpolation function and its first derivative $h^{\prime }\left(\xi \right)=30\xi^{2}{\left(1-\xi \right)}^{2}$ can make the term only active at the electrode/electrolyte interface. Substituting Eqs. 24–26 into Eq. 27 yields

(28) $$\begin{array}{ccc} \frac{\partial \xi }{\partial t} & = & -L_{\sigma }\left[g^{\prime }\left(\xi \right)-\kappa \nabla^{2}\xi +\frac{\partial f_{\text{els}}\left(\xi ,u\right)}{\partial \xi }\right]\\ & & -\;M_{\eta }h^{\prime }\left(\xi \right)i_{0,\text{ud}}\text{exp}\left[\frac{\left(\beta -\alpha_{m}\right)\Omega \sigma_{h}}{RT}\right]\\ & & \times \;\left\{\text{exp}\left[\frac{\left(1-\beta \right)F\eta_{\text{cta}}}{RT}\right]-\frac{c_{Li^{+}}}{c_{0}}\text{exp}\left(\frac{-\beta F\eta_{\text{cta}}}{RT}\right)\right\} \end{array}$$

The exchange current density at the undeformed state is regarded as a constant, then we define

(29) $$L_{\eta }=M_{\eta }i_{0,\text{ud}}$$

In particular, the potential of active Li stays at 0 V (set the working electrode as the grounding state), whereas the potential of dead Li keeps pace with the potential of electrolyte on the calculation (< 0 V). Therefore, a step function $f_{d}=f_{\text{step}}\left(-\phi_{l}/\phi_{d}\right)$ is introduced for recognizing the active state of Li, where $\phi_{d}$ is the reference potential. $f_{d}$ steps from 1 to 0 when $\phi_{l}$ is greater than $\phi_{d}$, corresponding to the change from active Li to dead Li. Substituting this criterion into Eq. 28, we get

(30) $$\begin{array}{ccc} \frac{\partial \xi }{\partial t} & = & -f_{d}L_{\sigma }\left[g^{\prime }\left(\xi \right)-\kappa \nabla^{2}\xi +\frac{\partial f_{\text{els}}\left(\xi ,u\right)}{\partial \xi }+\frac{\partial f_{\text{noise}}\left(\xi \right)}{\partial \xi }\right]\\ & & -\;f_{d}L_{\eta }h^{\prime }\left(\xi \right)\text{exp}\left(\frac{\left(\beta -\alpha_{m}\right)\Omega \sigma_{h}}{RT}\right)\left(e^{\frac{\left(1-\beta \right)F\eta_{\text{cta}}}{RT}}-\frac{c_{Li^{+}}}{c_{0}}e^{\frac{-\beta F\eta_{\text{cta}}}{RT}}\right) \end{array}$$

#### 2.1.3 Mass conservation

The electrolyte is assumed to be an ideal binary electrolyte, including solvents, cation (Li^+^), and anion (*A*^−^). The mass balance of the ionic species is expressed as

(31) $$\frac{\partial c_{i}}{\partial t}=-\nabla \cdot N_{i}+R_{i},\;i=Li^{+},\;A^{-}$$

$N_{i}$ is the mass flux and it is described as

(32) $$N_{i}=-D_{i}\nabla c_{i}-z_{i}c_{i}F\frac{D_{i}}{RT}\nabla \phi_{l}$$

where $D_{i}$ denotes the diffusion coefficient. On the right side of Eq. 32, the first term describes the diffusion transport and the second term where Nernst–Einstein relation is used describes the electro migration. $R_{i}$ represents a source or sink term caused by the electrochemical reaction. It only exists on the interface and can be given by Eq. 30. Coupling Eqs. 31 and 32, we can get

(33) $$\frac{\partial c_{Li^{+}}}{\partial t}=\nabla \cdot \left(D_{Li^{+}}\nabla c_{Li^{+}}+c_{Li^{+}}F\frac{D_{Li^{+}}}{RT}\nabla \phi_{l}\right)-c_{\text{Li}}\frac{\partial \xi }{\partial t}$$

(34) $$\frac{\partial c_{A^{-}}}{\partial t}=\nabla \cdot \left(D_{A^{-}}\nabla c_{A^{-}}-c_{A^{-}}F\frac{D_{A^{-}}}{RT}\nabla \phi_{l}\right)$$

The assumption of electroneutrality (i.e., $c_{A^{-}}=c_{Li^{+}}$) and constant diffusion coefficients for both anion and cation (i.e., $D_{A^{-}}=D_{Li^{+}}$) are employed for further mathematical simplification of Eq. 34.

#### 2.1.4 Charge conservation

The current conduction in the lithium metal electrode follows Ohm's law and charge conservation:

(35) $$i_{s}=-\sigma_{s}\nabla \phi_{s}$$

(36) $$\nabla \cdot i_{s}=-FR_{\text{Li}}$$

where $\sigma_{s}$ is the electric conductivity.

The current density in the electrolyte includes the sum of the flux of all charged species, which yields

(37) $$i_{l}=F\sum_{i}z_{i}N_{i}=-F\sum_{i}z_{i}D_{i}\nabla c_{i}-\frac{F^{2}}{RT}\nabla \phi_{l}\sum_{i}z_{i}^{2}D_{i}c_{i}$$

The concentration gradient can be ignored when the electrolyte is fully mixed or when the concentration is high. Ionic conductivity is defined by $\sigma_{l}=\frac{F^{2}}{RT}\sum_{i}z_{i}^{2}D_{i}c_{i}$. Thus, Eq. 37 can be written as

(38) $$i_{l}=-\sigma_{l}\nabla \phi_{l}$$

According to the current balance in the electrolyte, we can get

(39) $$\nabla \cdot i_{l}=FR_{\text{Li}}$$

The electron transfer at the electrode/electrolyte interface satisfies

(40) $$-i_{s}\cdot n=i_{l}\cdot n=i_{\text{loc}}$$

where n the normal direction.

As for the phase field model, Eqs. 36 and 39 are rewritten as

(41) $$\nabla \cdot \left(-\sigma_{\text{eff}}\nabla \phi_{s}\right)=-Fc_{\text{Li}}\frac{\partial \xi }{\partial t}$$

(42) $$\nabla \cdot \left(-\sigma_{\text{eff}}\nabla \phi_{l}\right)=Fc_{\text{Li}}\frac{\partial \xi }{\partial t}$$

Herein, $\sigma_{\text{eff}}=h\left(\xi \right)\sigma_{s}+\left[1-h(\xi )\right]\sigma_{l}$ is the effective electric conductivity.

#### 2.1.5 Mechanics

As the quasi-static assumption is employed, the mechanical equilibrium equation is given by

(43) ∇·σ=0

with

(44) $$\sigma =C_{ijkl}\varepsilon_{kl}^{E}$$

Herein, ${ɛ}^{E}={ɛ}^{T}-{ɛ}^{0}$, where ${ɛ}^{T}$ is the total strain and ${ɛ}^{0}$ is the inelastic strain. According to the small-deformation theory, we can get

(45) $$\varepsilon^{T}=1/2\left[{\left(\nabla u\right)}^{T}+\nabla u\right]$$

The hydrostatic stress and von Mises stress were used to visualize the stress evolution.

### 2.2 Numerical implementations

A system composed of a Li metal electrode and an electrolyte (solid) is simulated using a phase-field method on the COMSOL Multiphysics 5.5 platform. The size of the system is set as 30 × 50 μm. Detailed geometry and boundary conditions are described in Fig. S1. Herein, a constant current density condition is employed, i.e. the boundary current density *i*_applied_ is set to a given expression. A constraint for the current density on the upper boundary is applied:

(46) $$i_{\text{applied}}A=\int_{\partial S}i_{l}\cdot ndS$$

where A and ∂S denote the boundary area and the boundary, respectively. The electrolyte potential $\phi_{l}$ at the lower boundary is arbitrarily set to zero. The “boot-strapped” potential ensures that there is a unique solution but has no impact on the electrode kinetics. Corresponding parameters are listed in Table S2.

### 2.3 Experimental methods

The spherical and dendritic Li were formed in the electrolyte of 1.0 M LiTFSI dissolved in 1:1 v/v DOL/DME with 2% LiNO_3_ and the electrolyte of 1.0 M LiPF_6_ dissolved in 1:4 v/v FEC/DMC, respectively. The columnar Li was acquired by the methods proposed by Zhang et al. [34]. The morphology of electrodeposited Li was visualized by a scanning electron microscope (JSM 7401F, JEOL, Japan) operated at 3.0 kV.

## 3 Results and discussion

### 3.1 Stripping of spherical Li

Both inhomogeneous electrochemical reactions and mechanical effects contribute to the formation of dead Li. For the former, our previous study has built a phase field model for simulating dead Li formation under inhomogeneous Li stripping, revealing the relation between the polarization curve and the capacity loss [35]. For the latter, it is critical for practical batteries but there are few studies involved. Different from the deposition of Li, the stripping of Li itself is a stress-free process. Consequently, stress variation in the stripping process is related to cell configurations and pressure management systems. The two typical forms of pressure applied are the space constraint and the constant pressure. For example, prismatic and cylindrical batteries using metal materials as shells belong to the space constraint, representing a stress release process during stripping. In contrast, the constant pressure throughout the stripping process is presented in the case of pouch-type batteries with spring-weighted clamps. The resulting stress distribution will induce mechanical effects, including possible mechanical damage and the interplay between electrochemistry and mechanics.

We present an outline of mechanical effects on working Li metal anodes. Following the works of Ganser and Monroe et al. for the description of stress-coupled electrochemical reaction [21,36], the change of mechanical states results in the shift of energy states and thereby influences the local stripping rate at a Li electrode/electrolyte interface (Fig. 1a). The stress state of separators or solid electrolytes is assumed to do no impact on the energy state according to the experiments performed by Carmona et al. [37]. In this case, the shift in equilibrium potential $U_{\text{eq}}$ because of the mechanical deformation equals

(47) $$\Delta U_{\text{eq}}=-\frac{\Omega \sigma_{h}}{F}$$

where Ω is the molar volume of the Li and $\sigma_{h}$ the hydrostatic stress. The corresponding formulation of stress-coupled electrochemical reaction kinetics can be described by a modified Bulter-Volmer equation Eqs. 24 and 25. It is known that compressive stress can facilitate the anodic reaction (stripping) but impede the cathodic reaction (plating). How severely reaction rates are changed depends on the yield strength of Li metal with size effects (Fig. 1b, Table S1) [38]. For bulk Li with a yield strength of 1.0 MPa, the corresponding max equilibrium potential drop is 0.13 mV using Eq. 1, whereas for dendritic Li with a yield strength of 100 MPa, it can reach 13.0 mV. Therefore, the stress distribution can change the current distribution combined with the SEI rupture caused by mechanical damage. Different stress distributions in electrodeposited Li can lead to various situations during the stripping process (Fig. 1c). In the case where the stress is concentrated at the root, stripping velocity at the root (*i*_root_) is faster than that at the tip (*i*_tip_) due to the fracture of SEI and the change of equilibrium overpotential at the root, which eventually aggravates the formation of dead Li. In contrast, dead Li is evitable if the stress is concentrated at the tip. Such a stripping process with mechano-electrochemical coupling will be captured by the phase field model in this contribution.

**Fig. 2:**
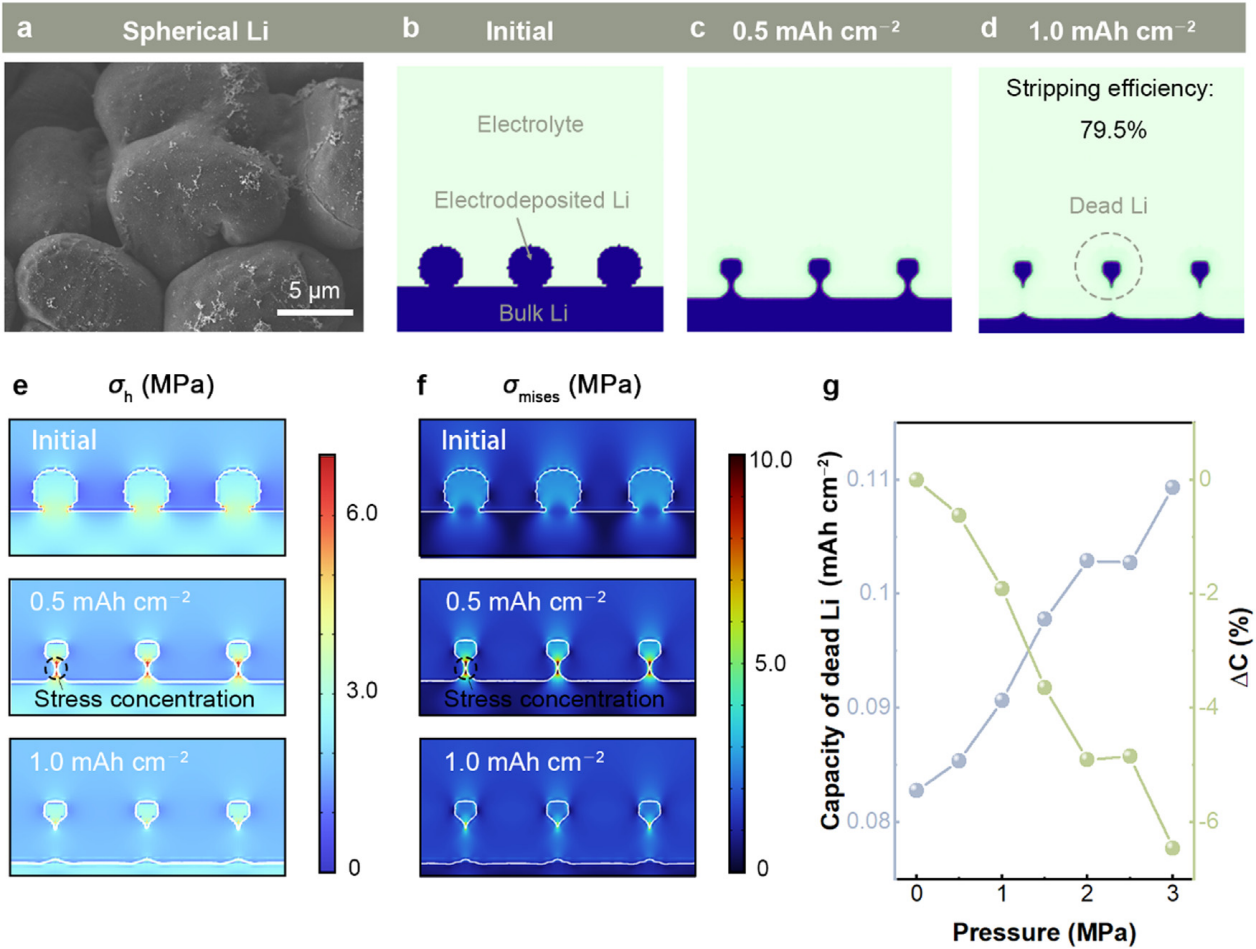
**The stripping of spherical Li*****.*** (a) Scanning electron microscope (SEM) images of spherical Li. (b–d) Simulated stripping process without external pressure. The simulated distribution of (e) hydrostatic stress *σ*_h_ and (f) von Mises stress *σ*_Mises_ under the external pressure of 3.0 MPa. (g) The specific capacity of dead Li and the deviation of stripping efficiency Δ*C* with respect to external pressure. The elastic modulus of the electrolyte is 1.0 GPa and the current density is fixed at 3.0 mA cm^−2^.

Spherical deposition morphology is a routine morphology in Li metal batteries with LiNO_3_ electrolyte additive (Fig. 2a). Such spherical geometry is simple, so we will carry out the stripping process based on this system at first. The simulation results of Li stripping with a stress-free field at a current density of 3.0 mA cm^−2^ are shown in Fig. 2b–d. As the stripping proceeds, the volume of the original spherical Li gradually becomes smaller and the bulk layer gradually thinner. A part of the electrodeposited Li loses electrical contact with the bulk layer at a stripping specific capacity of 0.6 mAh cm^−2^, thus becoming dead Li. The voltage, meanwhile, steps from 28 to 45 mV (Fig. S2). The stripping efficiency of spherical Li is 79.5%, implying a capacity loss of 20.5%. It is noted that the stripping efficiency of electrodeposited Li is distinguished from the Coulombic efficiency of batteries because bulk Li can compensate for the capacity loss of dead Li. Consequently, the generation of dead Li may not affect the overall Coulomb efficiency of a battery. The dead Li might be activated again or trouble the ion transport in the subsequent cycling process.

Corresponding concentration and potential distribution are shown in Fig. S3. Unlike the possible ion depletion nearby the electrode surface during plating, the Li^+^ concentration nearby the electrode surface during stripping is much higher than that in the bulk electrolyte, i.e., the electrochemical reaction at the surface is not restricted by mass transfer (Fig. S3a). Fig. S3b exhibits that the potential of the active Li is always at 0 V, while the dead Li remains consistent with the potential of the surrounding electrolyte. This indicates that the dead Li criterion can work well.

### 3.2 The influence of external pressure

A battery is restricted by metal shells (prismatic and cylindrical) or clamps (pouch cell) and experiences external pressure rather than a stress-free state in an actual test. Besides, the stress distribution is not necessarily uniform when the external pressure is transferred to the electrochemical reaction interface due to the mechanical properties and geometry of materials. Taking the external pressure of 3.0 MPa as an example, we simulated the distribution of hydrostatic stress (*σ*_h_) and von Mises stress (*σ*_Mises_) during the stripping process. The electrolyte elastic modulus (*E*_electrolyte_) is fixed at 1.0 GPa and the current density is set as 3.0 mA cm^−2^. As shown in Fig. 2e, the *σ*_h_ at the connection of spherical Li and bulk Li is always higher than other reaction locations before the formation of dead Li. According to Eq. 1, an increase in hydrostatic stress can lead to a negative shift in the equilibrium potential, increasing the power of Li stripping. On the other hand, the concentration of von Mises stress at the root shows a greater risk of SEI fracture, accelerating the root stripping as well (Fig. 2f). Therefore, the root stripping of spherical Li is accelerated compared to the stress-free state, contributing to an increase in the amount of dead Li.

Simulations of the dead Li formation as a function of external pressure are presented in Fig. 2g. When the external pressure increases from 0 to 3.0 MPa, the specific capacity of dead Li increases from 0.08 to 0.11 mAh cm^−2^, corresponding to the stripping efficiency of electrodeposited Li decreasing from 79.5% to 73.4%. The negative deviation of stripping efficiency (Δ*C*) is nearly linear with the increasing external pressure. Such a detrimental effect of external pressure on Li stripping is contrary to the plating process where external pressure can help reduce dendrite branches. Besides, it is noted that internal stresses (both *σ*_h_ and *σ*_Mises_) can far outweigh the applied external pressure (Fig. S4). The maximum values over the external pressure (range 0–3.0 MPa) go from 0 to 11.5 MPa for *σ*_h_ and 0–18.2 MPa for *σ*_Mises_. Consequently, estimating the mechano-electrochemical effect on Li stripping by the value of external pressure is inaccurate.

### 3.3 The influence of electrolyte properties and working conditions

**Fig. 3:**
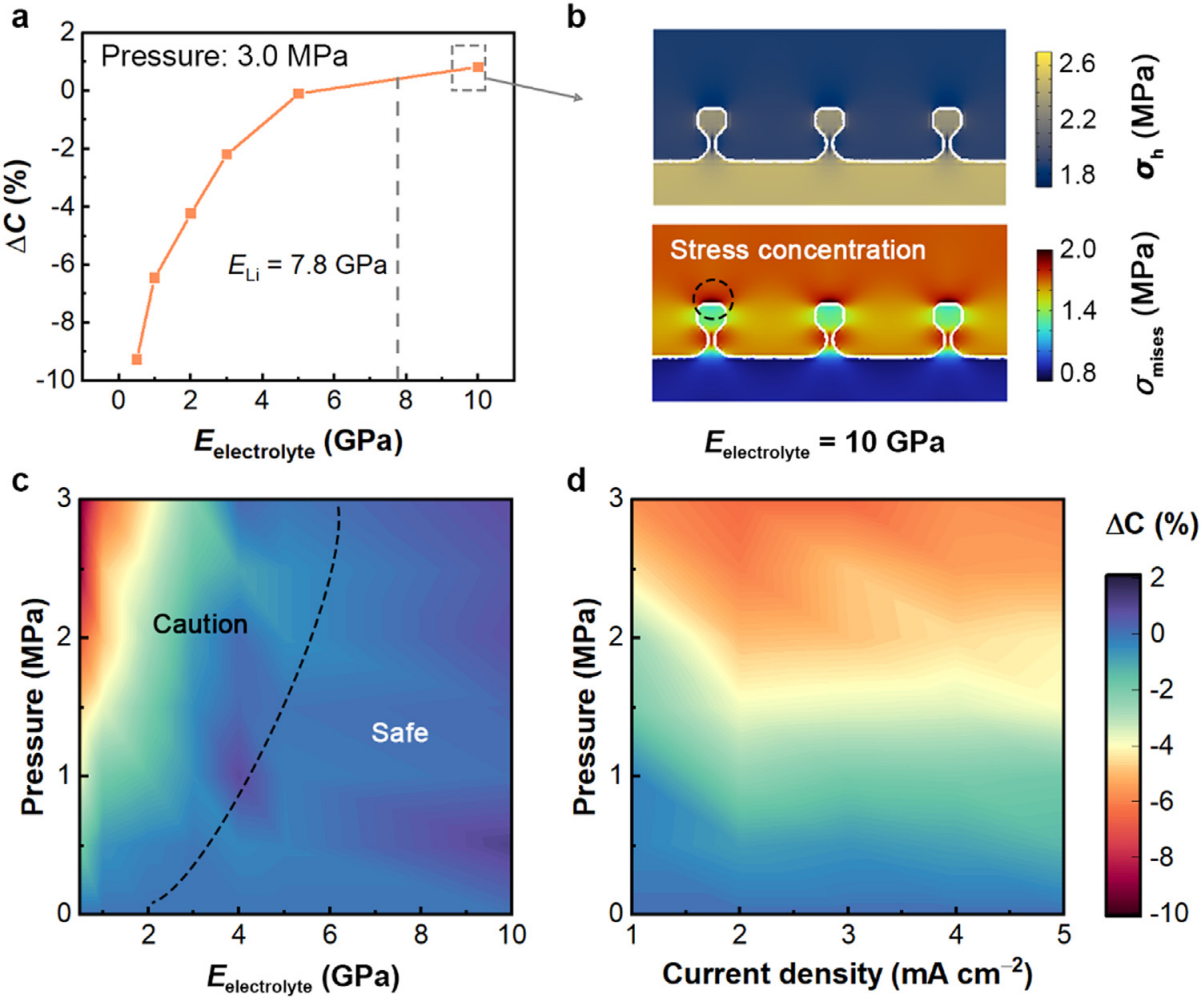
**The influence of electrolyte properties and working conditions.** (a) Contributions of 3.0 MPa pressure to Δ*C* as a function of electrolyte elastic modulus. (b) The simulated distribution of *σ*_h_ and *σ*_Mises_ when electrolyte elastic modulus is set as 10 GPa at a stripping specific capacity of 0.5 mAh cm^−2^. Contours of Δ*C* with respect to (c) applied pressure and electrolyte elastic modulus for a current density of 3.0 mA cm^−2^, (d) applied pressure and current density for an electrolyte elastic modulus of 1.0 GPa.

The interfacial stress stemming from the solid-solid contact is associated with the mechanical properties of electrolytes (or separators). Therefore, it is significant to extend our model to scenarios of various electrolytes, especially since increasing research is focusing on solid-state batteries [39]. Herein the external pressure is fixed at 3.0 MPa and *i*_applied_ = 3.0 mA cm^−2^, whereas *E*_electrolyte_ varied from 0.5 to 10.0 GPa. Fig. 3a shows that a higher *E*_electrolyte_ involves less dead Li formation, corresponding to increasing Δ*C*. Besides, a transition in Δ*C* from minus to plus occurs for *E*_electrolyte_ > *E*_Li_ (elastic modulus of Li), which implies that the effect of stress on dead Li formation has changed. The stress distribution under *E*_electrolyte_ = 10 GPa at a stripping specific capacity of 0.5 mAh cm^−2^ is captured in Fig. 3b. In comparison with *E*_electrolyte_ = 1.0 GPa, the location of stress concentration swift from root to others, leading to *i*_root_ < *i*_tip_. Consequently, external pressure at this point can help reduce the formation of dead Li. However, the interface problems, such as voids formation [40,41], will emerge with the increase of elastic moduli and current densities, which is beyond the scope of this model. A stripping model based on a quasi-static interface proposed by Shishvan et al. has brought light to the void formation [42].

High-throughput simulations were carried out for further analysis. The contours in Fig. 3c represent the deviation of stripping efficiency with respect to applied pressure and electrolyte elastic modulus for a current density of 3.0 mA cm^−2^. As the electrolyte modulus increases, the effect of external pressure on Δ*C* is weakened until reversed. In this regard, a dividing line (black dashed line in Fig. 3c) is proposed: For the caution region, the detrimental effect of external pressure on dead Li formation needs to be evaluated carefully; For the safe region, the externally applied pressure can hardly promote the dead Li formation.

To understand the influence of applied current densities, high-throughput simulations are included in Fig. 3d for a fixed electrolyte elastic modulus of 1.0 GPa. Applying a high current density can induce fast volume changes, i.e., fast stress concentration. Consequently, the effect of external pressure becomes more significant as the current density increases (0–2.0 mA cm^−2^). It is observed that Δ*C* is almost unchanged under a fixed external pressure when the current density is greater than 2.0 mA cm^−2^. Therefore, the effect of current density on dead Li formation becomes negligible.

### 3.4 Electrodeposited morphology dependency

**Fig. 4:**
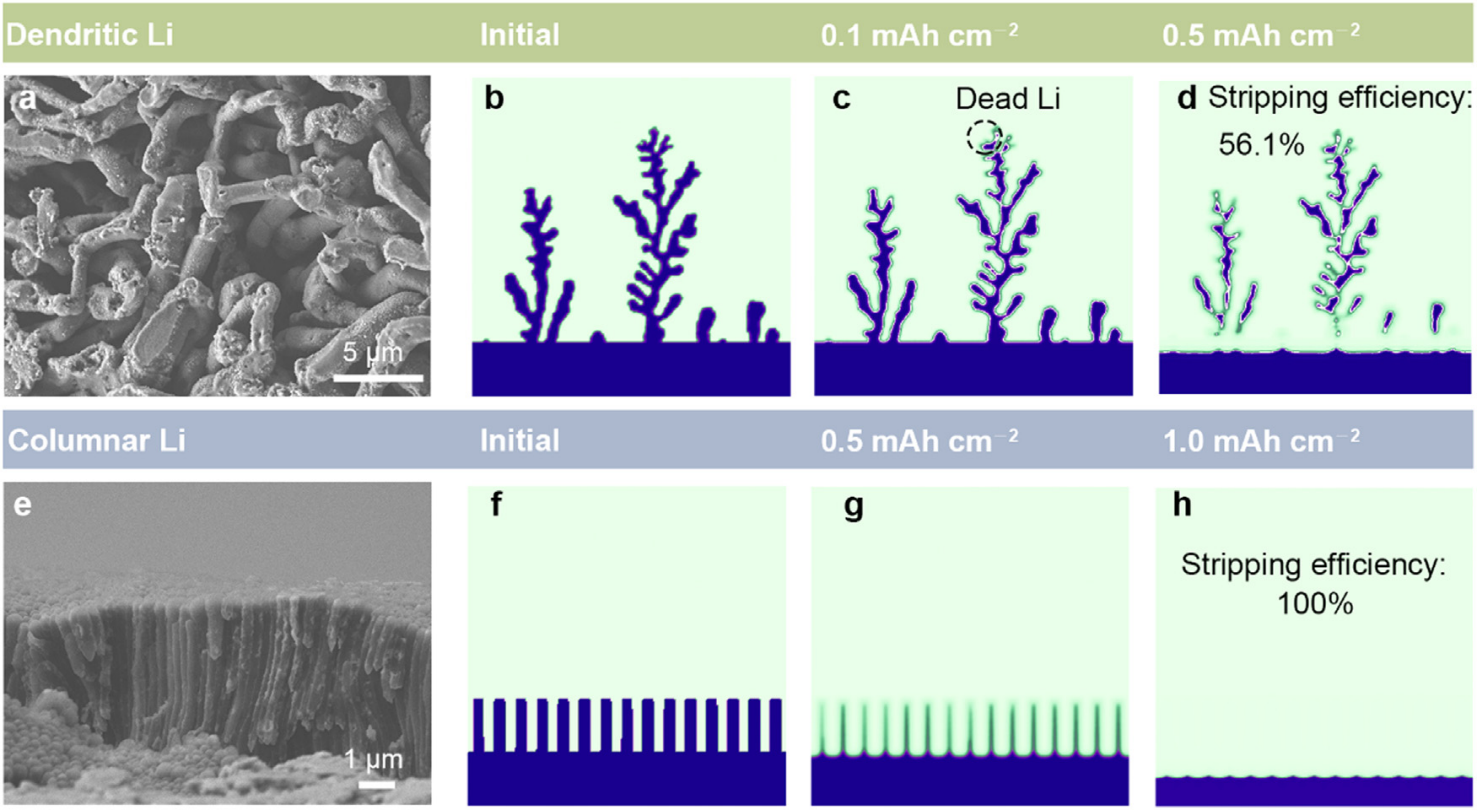
SEM images of (a) dendritic Li and (e) columnar Li. Simulating the stripping process of (b–d) dendritic Li and (f–h) columnar Li at a current density of 3.0 mA cm^−2^.

In addition to the common spherical deposition morphology, we further simulated the stripping process of irregular dendritic Li (Fig. 4a) and columnar Li (Fig. 4e). Stripping simulations of dendritic and columnar Li were carried out under the stress-free state for comparison with spherical Li, where the applied current density is 3.0 mA cm^−2^. Fig. 4b–d exhibits that the dendritic Li with many branching arms is prone to form dead Li, corresponding to a low stripping efficiency of 56.1%. In contrast, the columnar Li can strip thoroughly without dead Li formation. It is consistent with the observation of Nishikawa et al. [43] that the columnar Li is almost dissolved while the SEI shell remains. The above results indicate that the capacity of dead Li is closely related to the initial Li morphology and the stripping efficiency is column > sphere > dendrites. Theoretically, columnar Li is the most ideal morphology with the minimum formation of dead Li.

Applying an external pressure of 3.0 MPa, the stripping efficiency of dendritic Li reduces to 51.3%, while the stripping efficiency of columnar Li is 99.4%. It is to do with the stress distribution (Fig. S5). The complex morphology of dendritic Li tends to make local stress concentration, especially at the bifurcation (Fig. S5a and b), which promotes the formation of dead Li. The design of practical composite Li anode might draw lessons from such results, i.e., choosing vertically aligned anodes rather than porous anodes is beneficial to reducing dead Li formation [44,45].

### 3.5 Cell configuration and pressure management

The application of external pressure induces the increase of dead Li under *E*_electrolyte_ < *E*_Li_. Combined with the beneficial effect of external pressure in the plating process, such a double-edged effect from external pressure makes us rethink the cell configuration and pressure management of practical batteries. Consequently, the space-constrained configuration (e.g., prismatic cells)—where pressure is generated owing to volume expansion during charging (Li plating) but released during discharging (Li stripping)—is more advantageous than pouch cells under constant force.

## 4 Conclusion

A mechano-electrochemical phase-field model is proposed for simulating the Li stripping process, where both SEI rupture and equilibrium potential shift induced by stress are introduced into stripping kinetics. With the proposed model, the formation of dead Li with respect to external pressure under constant stripping current density is revealed: (1) The local concentration of stress at the root or bifurcation of electrodeposited Li, which is induced by the external pressure, promotes the formation of dead Li; (2) Such a detrimental effect of external pressure during stripping process can be alleviated by electrolytes with high electrolyte elastic modulus and vertically aligned electrodeposited morphology. Combined with the effect of external pressure on the plating process, segmented pressure management strategies for the charging and discharging process and space-constrained configuration for cell manufacture are appealed. This fundamental study provides new insights into dead Li formation under stress coupling in lithium metal batteries, which shed fresh light on the development of safe rechargeable batteries.

## Declaration of competing interest

The authors declare that they have no conflicts of interest in this work.

## Appendix Supplementary materials

Image, application 1
