# Supplementary material for: The dead lithium formation under mechano-electrochemical coupling in lithium metal batteries
Source: Fundam Res. 2022 Nov 23;4(6):1498–505. doi: 10.1016/j.fmre.2022.11.005 (PMC11670696; doi:10.1016/j.fmre.2022.11.005)
Supplement: Supplementary file 1 [file mmc1.pdf]

I. Supporting Text

Table S1. Mechanical properties of Li metal at room temperature

**Microstructure**	**Young's modulus (GPa)**	**Yield strength (MPa)**	**Test conditions**	**Ref.**
Single crystal, bulk <111>	21.2*	-	Acoustics	[1]
Single crystal, bulk <100>	3.0*	-	Acoustics	[1]
Single crystal, bulk	-	~0.2*	Tension	[2]
Single crystal, bulk (Diameter 1.0 cm)	-	~0.3*	Tension	[3]
Polycrystal rod (Diameter 1.27 cm)	7.82	0.73–0.81	Acoustics/Tension	[4]
Polycrystal rod (Diameter > 1.0 cm)	1.9	0.56	Compression	[5]
Polycrystal (Diameter 0.06–0.10 cm)	8.0	-	Acoustics	[6]
Polycrystal, bulk	7.8	0.76	Tension	[7]
Foil (Thickness, 750 μm)	7.82	-	Nanoindentation	[8]
Foil (Thickness, 750 μm)	9.43	0.57–1.26	Nanoindentation /Tension	[9]

Foil (Thickness, 18 μm)	8.2	-	Nanoindentation, 31°C	[10]
Foil (Thickness, 5 μm)	9.8	2.5–30*	Nanoindentation, 31°C	[10-11]
Pillars (0.98–9.45 μm)	-	15–105	Compression	[12]
Moss with a porosity of about 62.3%	1.6–2.6	-	Flat punch indentation	[8]
Whiskers（76–608 nm）	-	12–244	Compression	[13]
Dendrites（360–759 nm）	6.76±2.88	16±6.82	Compression	[14]

* Estimated based on references

Table S2. Physical and electrochemical parameters used in the model

Parameters	Value	Descriptions
L_σ	1×10^{-10} m^3 J^{-1} s^{-1}	Interfacial mobility
L_η	0.01 s^{-1}	Reaction constant
$i_{0,\text{ud}}$	20 A m^{-2}	The exchange current density at the undeformed state
κ_0	5×10^{-7} J m^{-1}	Gradient energy coefficient
δ	0.03	The strength of anisotropy
ω	4	A mode number of anisotropy
W	10^6 J m^{-3}	Barrier height
D_{Li^+}	1×10^{-11} m^2 s^{-1}	Diffusion coefficient of Li^+ in electrolyte
c_0	1000 mol m^{-3}	Initial concentration of electrolyte
c_{Li}	7.69×10^4 mol m^{-3}	Initial concentration of electrode
σ_s	1×10^7 S m^{-1}	Electrical conductivity of electrode
σ_l	1.0 S m^{-1}	Electrical conductivity of electrolyte
α	0.5	Charge transfer coefficients
E_{Li}	7.8 GPa	Young's modulus of electrode
E_e	1.0 GPa	Young's modulus of electrolyte

ν_{Li}	0.42	Poisson's ratio of electrode
ν_{e}	0.3	Poisson's ratio of electrolyte
C	10	Correction factor
ϕ_{d}	0.5 mV	Reference potential
T	298.15 K	Temperature

II. Supporting Figures

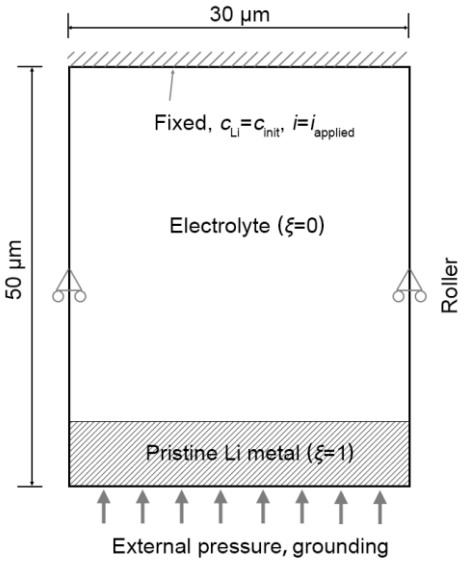

Fig. S1. The schematic of the simulated cell, including geometry and boundary conditions.

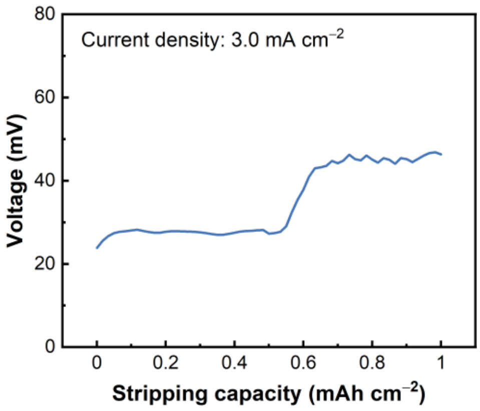

Fig. S2. The simulated voltage profile with respect to stripping capacity.

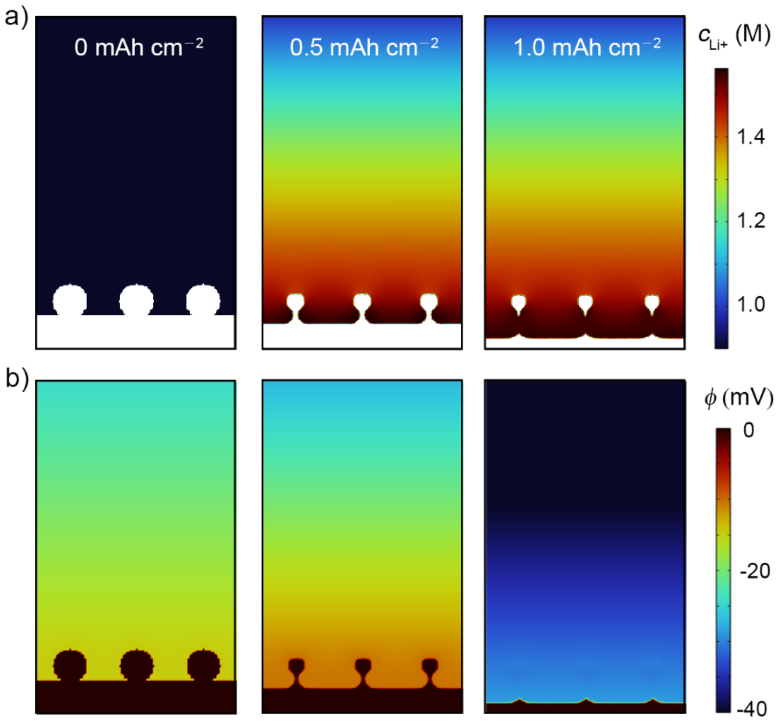

Fig. S3. Simulated distribution of (**a**) Li ion concentration and (**b**) potential.

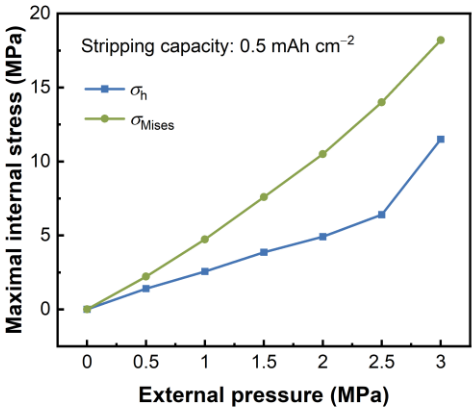

Fig. S4. Variation of maximal internal stress (hydrostatic stress and von Mises stress) with external pressure for a stripping capacity of 0.5 mAh cm^{-2}. All maximum values occur around the root.

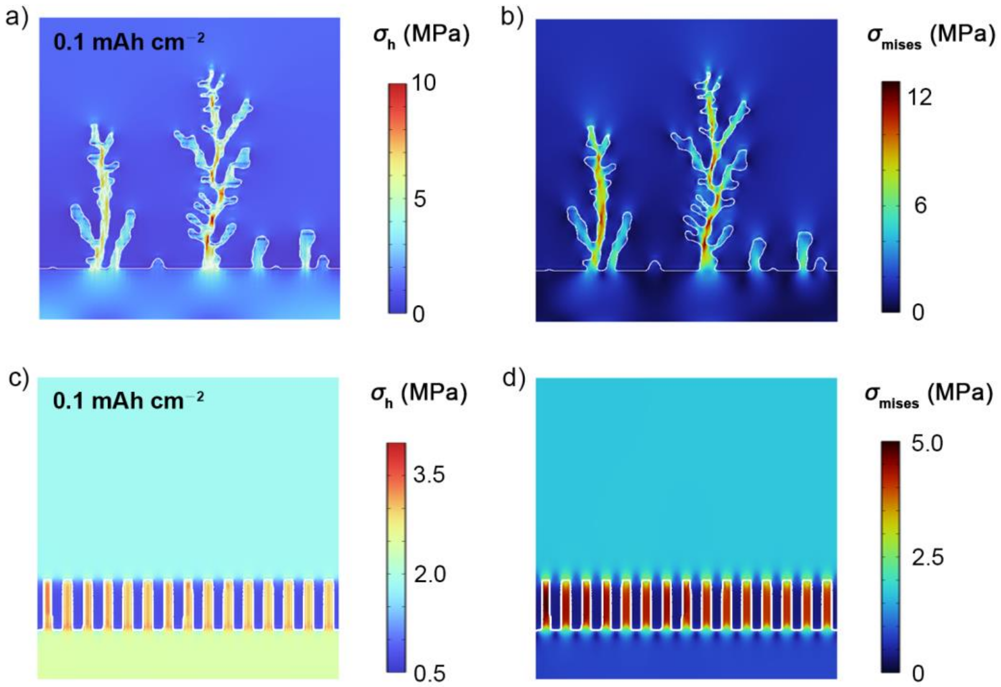

Fig. S5. The simulated stress distribution of (**a–b**) dendritic Li and (**c–d**) columnar Li under the external pressure of 3.0 MPa. The stripping specific capacity is 0.1 mAh cm^{-2} and the elastic modulus of electrolyte is 1.0 GPa.

References

[1] T. Slotwinski, J. Trivisonno, Temperature dependence of the elastic constants of single crystal lithium, J. Phys. Chem. Solids 30 (1969) 1276–1279.

[2] W. Pichl, M. Krystian, The flow stress of high purity alkali metals, Phys. Status Solidi A 160 (1997) 373–383.

[3] I. Gorgas, P. Herke, G. Schoeck, The plastic behavior of lithium single-crystals, Phys. Status Solidi A 67 (1981) 617–623.

[4] A. Masias, N. Felten, R. Garcia-Mendez, et al., Elastic, plastic, and creep mechanical properties of lithium metal, J. Mater. Sci. 54 (2018) 2585–2600.

[5] R. Schultz *Lithium: Measurement of Young's modulus and yield strength*; Fermi National Accelerator Laboratory: Batavia, 2002-10, 2002.

[6] W. M. Robertson, D. J. Montgomery, Elastic modulus of isotopically-concentrated lithium, Phys. Rev. 117 (1960) 440–442.

[7] S. Tariq, K. Ammigan, P. Hurh, et al. In *Li material testing—Fermilab antiproton source lithium collection lens*, Proceedings of the 2003 Particle Accelerator Conference.

[8] Y. K. Wang, D. Y. Dang, M. Wang, et al., Mechanical behavior of electroplated mossy lithium at room temperature studied by flat punch indentation, Appl. Phys. Lett. 115 (2019) 043903.

[9] C. D. Fincher, D. Ojeda, Y. Zhang, et al., Mechanical properties of metallic lithium: from nano to bulk scales, Acta Mater. 186 (2020) 215–222.

[10] E. G. Herbert, S. A. Hackney, N. J. Dudney, et al., Nanoindentation of high-purity vapor deposited lithium films: The elastic modulus, J. Mater. Res. 33 (2018) 1335–1346.

[11] E. G. Herbert, S. A. Hackney, V. Thole, et al., Nanoindentation of high-purity vapor deposited lithium films: A mechanistic rationalization of the transition from diffusion to dislocation-mediated flow, J. Mater. Res. 33 (2018) 1361–1368.

[12] C. Xu, Z. Ahmad, A. Aryanfar, et al., Enhanced strength and temperature dependence of mechanical properties of Li at small scales and its implications for Li metal anodes, Proc. Natl. Acad. Sci. USA 114 (2017) 57–61.

[13] L. Q. Zhang, T. T. Yang, C. C. Du, et al., Lithium whisker growth and stress generation in an in situ atomic force microscope–environmental transmission electron microscope set-up, Nat. Nanotech. 15 (2020) 94–98.

[14] M. A. Citrin, H. Yang, S. K. Nieh, et al., From ion to atom to dendrite: Formation and nanomechanical behavior of electrodeposited lithium, MRS Bull. 45 (2020) 891–904.
